# Natural products of traditional Chinese medicine treat atherosclerosis by regulating inflammatory and oxidative stress pathways

**DOI:** 10.3389/fphar.2022.997598

**Published:** 2022-09-30

**Authors:** Tianwei Meng, Xinghua Li, Chengjia Li, Jiawen Liu, Hong Chang, Nan Jiang, Jiarui Li, Yabin Zhou, Zhiping Liu

**Affiliations:** ^1^ Graduate School, Heilongjiang University of Chinese Medicine, Harbin, Heilongjiang, China; ^2^ Department of Pharmacy, Baotou Medical College, Baotou, Inner Mongolia, China; ^3^ Department of Cardiovascular Medicine, First Affiliated Hospital of Heilongjiang University of Chinese Medicine, Harbin, Heilongjiang, China; ^4^ Respiratoy Disease Department, First Affiliated Hospital of Heilongjiang University of Chinese Medicine, Harbin, Heilongjiang, China

**Keywords:** atherosclerosis, natural medicines, inflammatory factor, receptor, pathological mechanism

## Abstract

Atherosclerosis (AS) is a prevalent arteriosclerotic vascular disease that forms a pathological basis for coronary heart disease, stroke, and other diseases. Inflammatory and oxidative stress responses occur throughout the development of AS. Treatment for AS over the past few decades has focused on administering high-intensity statins to reduce blood lipid levels, but these inevitably damage liver and kidney function over the long term. Natural medicines are widely used to prevent and treat AS in China because of their wide range of beneficial effects, low toxicity, and minimal side effects. We searched for relevant literature over the past 5 years in databases such as PubMed using the keywords, “atherosclerosis,” “traditional Chinese medicine,” “natural medicines,” “inflammation,” and “oxidative stress.” We found that the PI3K/AKT, TLR4, JAK/STAT, Nrf2, MAPK, and NF-κB are the most relevant inflammatory and oxidative stress pathways in AS. This review summarizes studies of the natural alkaloid, flavonoid, polyphenol, saponin, and quinone pathways through which natural medicines used to treat AS. This study aimed to update and summarize progress in understanding how natural medicines treat AS *via* inflammatory and oxidative stress-related signaling pathways. We also planned to create an information base for the development of novel drugs for future AS treatment.

## Introduction

Atherosclerosis (AS) is a chronic progressive inflammatory disease (Zhu et al., 2018). It is the main pathological basis of cardiovascular and cerebrovascular diseases such as coronary heart disease, myocardial infarction, acute coronary syndrome, and stroke (Marchio et al., 2019). Statistics have indicated that the global population of patients with carotid plaque has increased from 513 to 816 million between 2000 and 2023, and the prevalence of carotid stenosis has increased by 59.13% (Song P. et al., 2020). This is due to population aging, smoking, unhealthy dietary habits, and other factors. According to a recent report from the World Health Organization (WHO), cardiovascular disease remains the leading cause of death worldwide (GBD 2017 DALYs and HALE Collaborators, 2018). Stabilizing and reversing plaque are considered important strategies for treating atherosclerotic cardiovascular disease (ASCVD), and anti-inflammatory and antioxidant strategies have also become mainstream (Steven et al., 2019).

Inflammation and oxidative stress are the important pathological features of ASCVD (Yuan et al., 2019). The occurrence and development of inflammation are closely associated with oxidative stress. Abnormal vasodilation caused by low-density lipoprotein (LDL) produced during oxidative stress is an initiating factor in the inflammatory response of AS. Simultaneously elevated levels of oxidative stress and inflammatory responses mediate damage to the vascular endothelium, which recruits monocytes to differentiate into macrophages, absorbs oxidized low-density lipoprotein (ox-LDL) and slowly transforms into foam cells, which are early signs of AS formation (Varghese et al., 2018; Marchio et al., 2019; Poznyak et al., 2020). Foam cells further propagate and amplify the inflammatory response, stimulating platelet aggregation and adhesion to damaged vascular endothelium. This further promotes the formation of AS plaques.

The inflammatory and oxidative stress responses involved in AS development are associated with abnormalities in several signal transduction pathways. The abnormal regulation of various signaling pathways in the vascular intima leads to atypical expression of inflammatory factors and proinflammatory mediators. These induce the continuous production of reactive oxygen species (ROS) and oxidative stress, and finally forms AS. This article reviews the known inflammatory and oxidative stress-related, phosphatidylinositol 3-kinase/protein kinase B (PI3K/AKT), Toll-like receptor 4 (TLR4), Janus kinase signal transducer and activator of transcription (JAK/STAT), nuclear factor erythroid 2–related factor 2 (Nrf2), mitogen-activated protein kinase (MAPK), and nuclear factor kappa-light-chain-enhancer of activated B cell (NF-κB) signaling pathways that are involved in the pathological process of AS. The structure and function of these pathways and their roles in AS pathogenesis are emphasized herein.

Antiplatelet drugs and statins are currently the most prevalent drugs for the clinical treatment of ASCVD (Kishore et al., 2018). Anakinra and canakinumab both block interleukin (IL)-1β, and tocilizumab blocks IL-6 that are inflammatory factors involved in the occurrence and development of AS and synthetic antioxidants such as probucol reduced cholesterol levels (Chistiakov et al., 2018; Gluba-Brzózka et al., 2021). However, the high cost and side effects of long-term drugs presently on the market and synthetic drugs under development have clarified that suitable alternatives are urgently needed. Traditional Chinese medicine (TCM) has a history of thousands of years, and it plays a key role in the prevention and treatment of cardiovascular diseases. Natural medicines including TCM have the advantages of low toxicity and side effects and are widely applicable. However, TCM also has the characteristics of multiple components and targets that function concurrently or synergistically in the treatment of diseases. However, the complex mechanisms of these components and targets are difficult to fully explain, and systematic reviews of the relevant pathways of natural products with which to treat AS are scant. We searched the PubMed, SciFinder, and Web of Science databases. Here, we summarize the inflammatory and oxidative stress signaling pathways involved in the pathological process of AS, and systematically review current progress in natural products that can treat AS through acting on these pathways. The advantages and disadvantages of the experimental design of natural TCM products acting on AS is discussed, and in-depth insights are provided for followup investigation.

## Classical signaling pathway

**FIGURE 1:**
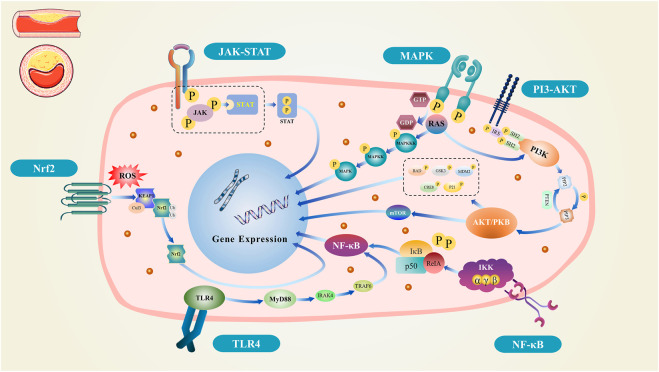
Schematic diagram of the signal pathway.

The occurrence and development of AS are affected by processes such as the inflammatory response, oxidative stress, hemodynamics, cholesterol metabolism, angiogenesis, and foam cells. Abnormal activation of different signaling pathways directly or indirectly interferes with different AS stages. Here, we summarize the inflammatory and oxidative stress pathways involved in AS. Understanding the factors and pathways that contribute to AS pathology can inform future experimental and clinical studies. Figure 1 shows a schema of the signaling pathways.

### PI3K/AKT signaling pathway

The signaling pathway consists of PI3K and its downstream molecule, PKB/AKT (Qin et al., 2021). Various extracellular signals trigger intracellular P13K to activate PKB/AKT, and AKT is a major downstream molecule of the PI3K signaling pathway (Zhao et al., 2021) that functions in promoting metabolism, proliferation, survival, growth, and angiogenesis. Activation of the PI3K/AKT signaling pathway inhibits the expression of proinflammatory factors caused by NF-κB and transforms the phenotype of microglia from pro, to anti-inflammatory. This activated pathway inhibits neuroinflammation mediated by proinflammatory microglia and attenuates inflammatory responses (Xu et al., 2018). In contrast, activated AKT further activates Nrf2, prevents Kelch-like ECH-associated protein 1 (Keap1) from coupling with it, promotes antioxidant response element (ARE) binding to it, and enhances the expression of antioxidant proteins (Li J. et al., 2021).

Phosphatidylinositol 3-kinase/protein kinase B (PI3K/AKT) signaling pathways mainly influence the development of AS by regulating inflammatory immunity and chemokines. The PI3K/AKT signaling pathway mediates dendritic cells to intervene in immune responses, thereby initiating inflammatory immunity (Monaci et al., 2021). Chemokines are also affected by the PI3K/AKT signaling pathway, prompting inflammatory cell activation and the regulation of macrophage polarization. The activation of inflammatory cells is significantly attenuated or disappears in macrophages without PI3K. Macrophages with AKT2-knockout have decreased levels of inflammatory factors, decreased cell migration ability, and foam cell formation, as well as increased cholesterol efflux. This leads to macrophage polarization towards the M2 type, which enhances plaque stability (Graves and Milovanova, 2019). The PI3K/AKT signaling pathway is also involved in lipid metabolism in endothelial cells. Inhibiting the PI3K/AKT signaling pathway reduces lipid deposition in vascular smooth muscle cells (VSMCs), increases lipid efflux, improves blood lipid metabolism disorders, and reduces the probability of plaque formation (Pi et al., 2021). Activation of the PI3K/AKT signaling pathway inhibits VSMC migration and reduces platelet adhesion. These results are attributed to the regulation of many important platelet responses by PI3K and its downstream effector AKT, such as changes in platelet shape, irreversible platelet aggregation, and increased thrombus volume (Sun et al., 2018).

### TLR4 signaling pathway

As an important pattern recognition receptor of the Toll-like receptor (TLR) family involved in the inflammatory response, TLR4 is distributed in vascular endothelial cells, neutrophils, mononuclear macrophages, pancreatic islet endocrine cells, cardiomyocytes, and dendritic cells. All of these cells can form a bridge between innate and acquired immunity (Vijay, 2018). After TLR4 binds to specific ligands, it transmits stimulatory signals to the nucleus by activating a series of downstream protein cascades. This series of signal transductions promotes the activation of various immune response genes namely, nuclear factor kappa B (NF-κB), transcription factor complex activator protein-1 (AP-1), and interferon regulatory factors (IRFs) and induces the expression of various cytokines and adhesion molecules associated with the inflammatory response. This has become recognized as a pathogenic mechanism of AS (Kim et al., 2018). Oxidative stress and cellular damage activate TLR4, leading to the expression of inflammatory mediators (Zhu et al., 2021).

Toll-like receptor 4 (TLR4) has numerous ligands that are involved in the formation and development of AS. Lipopolysaccharide (LPS) upregulates the expression of lectin-like oxidized low-density lipoprotein receptor-1 (LOX-1) through the TLR4 signaling pathway, prompting macrophages to engulf oxidized lipids and convert them into foam cells that aggregate and increase plaque areas. In addition, LPS is involved in degradation of the extracellular matrix and weakening of the fibrous cap, which increases plaque instability and promotes plaque rupture (Jongstra-Bilen et al., 2017; Ciesielska et al., 2021). Oxidized LDL upregulates matrix metalloproteinase-9 (MMP-9) through the TLR4 signaling pathway, prompting macrophages to express a series of inflammatory factors and accelerate plaque rupture (Li T. et al., 2021). Furthermore, TLR4 downregulates the expression of ATP-binding cassette transporter G (ABCG1) and induces lipid accumulation and the infiltration of inflammatory cells into vascular smooth muscle, thereby mediating AS formation (Cao et al., 2018). Therefore, as a mediator of lipid metabolism, immune response, and chronic inflammation, a TLR4 deficiency significantly reduces the expression of proinflammatory factors while reducing lipid and macrophage components in AS plaques.

### JAK/STAT signaling pathway

The Janus kinase-signal transducer and activator of transcription (JAK/STAT) signaling pathway is an essential multifunctional cascade of cytokine and growth hormone receptor signals (Owen et al., 2019). It is involved in regulating gene expression and cellular physiological processes. Janus kinase binds to extracellular transmembrane receptors and cytokines, consequently promoting JAK activation and tyrosine phosphorylation. Activated JAK binding to its receptor activates signal transducers and transcriptional activators in the cytoplasm, resulting in the tyrosine phosphorylation of STAT and the formation of homologous or heterologous dimers in the nucleus. Janus kinase then binds to the DNA sequence of the target gene promoter in the nucleus for specific gene expression (Herrera and Bach, 2019; Xu et al., 2020).

The JAK/STAT signaling pathway can mediate AS formation from various aspects, such as vascular endothelial cell (VEC) dysfunction, vascular smooth muscle (VSMC) proliferation and migration, and inflammatory cell infiltration (Hossain et al., 2021). The activation, proliferation, and migration of endothelial cells are the basis for the generation of new blood vessels, and immature new blood vessels can lead to AS plaque instability and even rupture. The proliferation and migration of endothelial cells is completed by the induction of vascular endothelial growth factor (VEGF), and the JAK/STAT signaling pathway is the main pathway of intracellular VEGF signaling (Bernal et al., 2021). Inhibiting this pathway can reverse VSMC proliferation and migration into a static state and inhibit the synthesis of many inflammatory mediators. Additionally, JAK/STAT signaling molecules are have been identified in AS plaques and inflammation-stimulated vascular cells. A lack of STATs in vascular endothelial cells or inflammatory cells can inhibit AS plaque formation (Tang et al., 2020). Inhibiting the JAK/STAT signaling pathway can antagonize LPS-induced AS (Hashimoto et al., 2020). Suppressors of cytokine signaling (SOCS) in the JAK/STAT pathway reduce STAT activity and inhibit subsequent proinflammatory responses. Moreover, SOCS can combine with various inflammatory factors to limit the inflammatory response of the vascular intima, thus inhibiting the occurrence of AS (Lopez-Sanz et al., 2018).

### Nrf2 signaling pathway

The nuclear transcription factor Nrf2 contains seven domains (Neh1, Neh2, Neh3, Neh4, Neh5, Neh6 and Neh7) and is ubiquitous in organs that consume oxygen. It regulates various antioxidant protein genes (Alam et al., 2017) and the activities of various antioxidant enzymes through the antioxidant damage pathway. These antioxidant enzymes protect cells by reducing oxidative stress damage and inflammatory responses in several ways (He et al., 2020).

As a central regulator of intracellular redox homeostasis, the Nrf2 signaling pathway maintains a balanced intracellular redox process, which is crucial for anti-AS as well as cardiovascular and cerebrovascular protection (Chen and Maltagliati, 2018). Deletion Nrf2 or its disordered activation aggravates the cytotoxicity of oxidative stressors, cause oxidative damage to the vascular wall, and eventually leads to AS (Alonso-Piñeiro et al., 2021). The formation of AS not only damages the arterial vascular endothelium, but also destabilizes homeostasis of the intracellular milieu (Saha et al., 2020). Furthermore, lipid accumulation in the arterial intima recruits numerous inflammatory cells to the vascular endothelium, leading to the sustained production of cytokines and reactive oxygen species (ROS). The Nrf2 signaling pathway protects vascular endothelial cells from oxidative stress damage, participates in the regulation of macrophages, reduces intracellular lipid accumulation, and inhibits foam cell formation (Robledinos-Antón et al., 2019). Therefore, the Nrf2 signaling pathway exerts antioxidant action by protecting the vascular endothelium, reducing lipid accumulation, and inhibiting inflammation, thus diminishing AS development.

### MAPK signaling pathway

The important MAPK signaling pathway mediates extracellular signals and reaction information transmission between cells and nuclei. This pathway mainly comprises extracellular signal-regulated kinase (ERK), c-Jun N-terminal protein kinase (JNK), and p38 mitogen-activated protein kinase (p38MAPK) (Zhang L. et al., 2019). The hub of this signaling pathway is MAPK that when activated, phosphorylates nuclear transcription factors, cytoskeletal proteins, and enzymes. Activation of the MAPK signaling pathway is associated with the release of various inflammatory cytokines and oxidative stress and it is mainly involved in the cellular inflammatory response and apoptosis under stress (Guo et al., 2021).

Atherosclerosis is closely associated with activation of the MAPK signaling pathway. This pathway is affected by oxidative stress-mediated endothelial dysfunction and the expression of proinflammatory factors that affect the occurrence and development of AS. When the MAPK signaling pathway is activated by ox-LDL, MAPK phosphorylation in the blood produces abundant ROS, which promote the MAPK signaling pathway to induce monocyte accumulation in the arterial wall, reduce the secretion of collagen and other matrices by vascular smooth muscle cells, and elicit cytotoxicity (Gong et al., 2019). This leads to foam cell necrosis in vascular plaque, resulting in AS plaque fragmentation and the eventual formation of thrombus in blood vessels. Therefore, blocking the ROS/MAPK signaling pathway is one mechanism through which oxidative stress-mediated endothelial dysfunction is alleviated and foam cell formation is prevented (Geng et al., 2018; Fang et al., 2021). The activation of MAPK is driven by inflammation. Stimulation of intermittent hypoxia/reoxygenation (IHR) can activate the MAPK pathway in endothelial cells and induce the expression of proinflammatory cytokines (Ryan et al., 2007). Conversely, specific MAPK inhibitors reduce the IHR-induced expression of proinflammatory factors. Intervention in the MAPK signaling pathway can also modulate the degree of intimal proliferation, platelet activation, and apoptosis (Jeon et al., 2021), all of which are key factors in AS formation.

### NF-κB signaling pathway

The NF-κB transcription factor family comprises homodimeric or heterodimeric subunits of the Rel family, including Rel (p65), NF-κB1 (p50), NF-κB2 (p52), RelB, and c-Rel. Activation of the IκB kinase (IKK) complex initiates NF-κB pathway signals (Mulero et al., 2019; Poma, 2020). Under the action of inflammatory factors, NF-κB, which is a normal cytoplasmic component, is transferred to the nucleus where it binds to the κB sequence in the promoter region of the relevant target gene sequence and induces the expression of proinflammatory cytokines, adhesion molecules, chemokines, matrix metalloproteinases, and acute-phase proteins associated with inflammation (Mitchell and Carmody, 2018).

Nuclear factor-kappa B is the most widely studied key regulator of inflammatory responses, as it participates in the initiation and development of AS lesions and plays a key role in the disease process. It participates in all stages of AS, from plaque formation to rupture, by regulating the activation of endothelial cells and the expression of proinflammatory factors (Zhong et al., 2018). Nuclear factor-kappa B accelerates the growth of lipid plaques and promotes inflammatory responses by regulating the expression of cytokines such as IL-1β, tumor necrosis alpha (TNF-α), and interferon gamma (INF-γ) (Chen et al., 2018). The NF-κB signaling pathway regulates the invasion and colonization of inflammatory cells in vascular walls, changes the composition of the extracellular matrix, and promotes the migration of smooth muscle cells (Zhang Q. et al., 2021). Nuclear factor-kappa B is also an oxidative stress-sensitive transcription factor. Intracellular ROS directly mediate the upregulation of NF-κB. After IκB is phosphorylated, activated NF-κB enters the nucleus and initiates inflammatory responses (Wu et al., 2018a). These directly lead to the inflammatory and pro-apoptotic responses of vascular endothelial cells, which drive the progression of AS.

## Natural medicines for atherosclerosis

**FIGURE 2:**
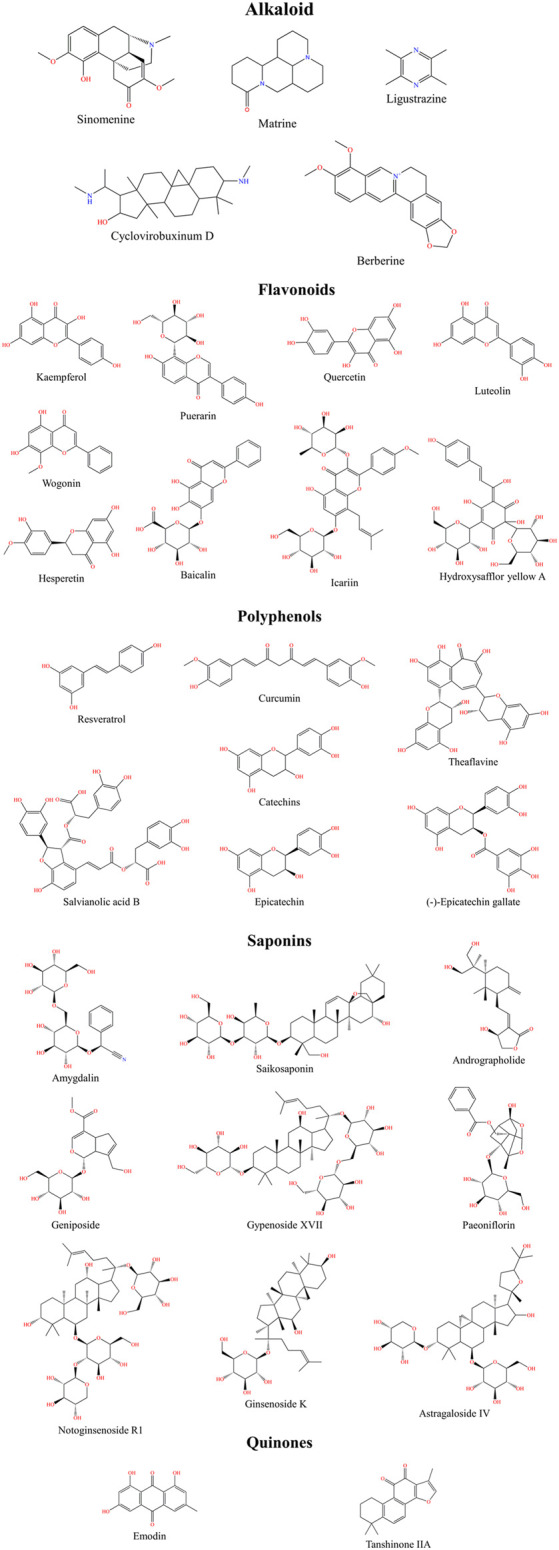
Natural medicine chemical structural formula.

Traditional Chinese Medicines are natural and offer unique advantages for treating cardiovascular diseases. They regulate lipid metabolism, resist lipid peroxidation, inhibit the proliferation and migration of VSMCs, are anti-inflammatory and anticoagulant, and protect vascular endothelial cells. TCMs can block the occurrence and development of AS through different signaling pathways. Therefore, we reviewed the effects and mechanisms of various natural medicines on AS from the perspectives of anti-inflammatory and antioxidant properties. Figure 2 shows the chemical structure of a natural medicine.

### Alkaloids

Alkaloids are a ubiquitous class of organic compounds containing nitrogen and have complex cyclic structures, and alkaline properties (Ghorbani et al., 2021). Alkaloids are divided structurally into piperidines, isoquinolines, quinolone alkaloids, indoles, terpenes, and steroids. Alkaloids are the active components of many Chinese herbal medicines, due to their powerful antitumor, anti-inflammatory, antibacterial, antiviral, and insecticidal properties. Some of them have been applied to treat cardiovascular diseases (Liu et al., 2019). For example, Cyclovirobuxinum D extracted from the TCM plant *Buxus sinica var. Parvifolia* M. Cheng can protect blood vessels and the heart by activating the JAK/STAT signaling pathway, reducing nitric oxide (NO) release and intracellular NO (iNOS) expression, and inducing macrophages to participate in the initiation and regulation of immune inflammation (Guo et al., 2014). Matrine inhibits the NF-κB/MAPK signaling pathway and the production of ROS in human aortic smooth muscle cells (HASMCs), which subsequently decreases the expression of inflammatory TNF-α(Wang et al., 2021). Matrine can inhibit activation of the TLR4 signaling pathway, promote macrophage polarization towards M2, and reduce the expression of elevated DNA methyltransferase in macrophages. This further inhibits oxidative stress, which slows AS progression (Cui et al., 2022). Sinomenine extracted from the TCM *Sinomenium acutum* (Thunb.)Rehder&E.H.Wilson regulates lipid metabolism, reduces intracellular cholesterol levels, alters TLR4 and NF-κB signaling pathways, downregulates the expression of inflammatory factors, and inhibits oxidative stress and vascular inflammation (Geng et al., 2021). Ligustrazine significantly expression TLR4 inhibits in blood vessels (Yang L. et al., 2021) and block the activation and nuclear translocation of NF-κB and Nrf2(Li Y. et al., 2019), consequently inhibiting monocyte adhesion to the endothelium. Ligustrazine simultaneously affects platelet aggregation, secretion, and adhesion by inhibiting the PI3K/AKT pathway, thus exerting an anti-platelet activation effect (Li L. et al., 2019). Berberine promotes intimal growth repair and reduces or stabilizes plaques, which is associated with mobilizing arterial intimal inflammatory and immune factors to regulate the PI3K/AKT signaling pathway (Song and Chen, 2021).

### Flavonoids

Many plants produce flavonoids as secondary metabolites. Flavonoids have a benzo-γ-pyranone parent nucleus and comprise isoflavones, flavanones, flavanols, and cyanidin chlorides according to their chemical structures (Badshah et al., 2021; Wen et al., 2021). Flavonoids have antioxidant, anti-inflammatory, and anticancer properties, and protect the cardiovascular system (Ciumărnean et al., 2020). Kaempferol reduces ox-LDL and inhibits intracellular ROS production Therefore, activation of the G protein-coupled estrogen receptor (GPER) and PI3K/AKT/Nrf2 pathways is enhanced, which in turn inhibits inflammation and apoptosis, reduces lipid levels and restores vascular morphology (Feng et al., 2021). Quercetin blocks expression of the NF-κB signaling pathway by inhibiting the upregulation of VCAM-1, ICAM-1, and E-selectin in HASMCs. It prevents the chemotaxis of endothelial cells to monocytes and their adhesion, protects endothelial cells, and delays the occurrence and development of AS (Chen et al., 2020). Puerarin is a hydroxyisoflavonoid extracted from the TCM *Puerariae Lobata* (Willd.) Ohwi. It activates the PI3K/AKT/eNOS signaling pathway and simultaneously inhibits the NF-κB signaling pathway, resulting in downregulated tissue factor (TF) expression in human umbilical vein endothelial cells (HUVECs), and ultimately inhibiting thrombosis (Deng et al., 2017). Icariin inhibits ROS generation by inhibiting the NF-κB/MAPK signaling pathway and upregulating the activity of the Nrf2 signaling pathway, thus inhibiting the transcription of cell adhesion factors and chemokines. This mechanism inhibits the inflammatory response and antioxidative stress by significantly reducing the expression of factors and inhibiting plaque formation in blood vessels (Hu et al., 2016; Luo et al., 2022). Luteolin exerts anti-AS effects by downregulating expression of the cytokines VCAM-1 and TNF-α, thus reducing oxidative stress and inflammatory response. This is associated with its inhibition of the TNF-α-mediated feedback system and downregulation of the NF-κB/MAPK pathway (Xia et al., 2014; Jia et al., 2015). The main extract of the TCM *Carthamus tinctorius* L. is Hydroxysafflower yellow A, which inhibits the formation of foam cells, reduces the inflammation of blood vessel walls, maintains the integrity of the structure and function of endothelial cells, and inhibits platelet aggregation. These mechanisms are achieved by regulating the TLR4 signaling pathway, oxidative stress, and lipid metabolism (Xue et al., 2021). Baicalin (Wu et al., 2018b), wogonin (Wu et al., 2019), and hesperitin (Olumegbon et al., 2022) inhibit plaque formation *via* anti-inflammatory and anti-oxidative stress mechanisms, and play roles in protecting blood vessels.

### Polyphenols

Polyphenolic compounds are a diverse group of plant components with several phenolic hydroxyl groups in their molecular structures, including flavonoids, tannins, phenolic acids, and anthocyanins (Singla et al., 2019). Polyphenols have anti-inflammatory and antioxidant capabilities that help to treat cardiovascular diseases (Fraga et al., 2019). Resveratrol is a natural cardioprotective polyphenol (Cheng et al., 2020) that acts on many of the pathways described herein to inhibit lipid peroxidation, antiplatelet aggregation, and inflammation (Pan et al., 2016; Zhang M. et al., 2019; Chen et al., 2022; Ji et al., 2022). Curcumin, extracted from *Curcuma longa* L, downregulates levels of proteins associated with the NF-κB signaling pathway by inhibiting the synthesis of cellular proteins required for cytomegalovirus replication. This results in proinflammatory cytokine inhibition and enhanced antioxidant enzyme activity. Curcumin also reduces lipid deposition and cardiovascular inflammatory damage (Lv et al., 2020). Theaflavine increases the activity of various antioxidant enzymes, reduces the activity of monoamine oxidases (MAO) and the lipid peroxide malondialdehyde (MDA), and upregulates the Nrf2 signaling pathway in vascular endothelial cells. This mechanism protects HUVECs from cholesterol-induced oxidative damage and inhibits AS plaque formation and changes in aortic histology (Zeng et al., 2021). Salvianolic acid B attenuates ox-LDL or LPS-induced inflammatory cytokines in cells and improves lipid deposition in the aorta. This is achieved by inhibiting phosphorylation of the MAPK/NF-κB signaling pathway (Zhang et al., 2022b). Catechin (Starzonek et al., 2019), epicatechin (Morrison et al., 2014) and (-)-epicatechin gallate extracted from the leaves of *Camellia sinensis* (L.) Kuntze help to stabilize AS plaques through the NF-κB signaling pathway and (-)-epicatechin gallate regulates lipid metabolism and improves foam cells through the MAPK/TLR4 signaling pathway (Li W. et al., 2021).

### Saponins

Triterpenoid and steroidal saponins are glycosides that are ubiquitous in plants and have antitumor, anti-inflammatory, immunomodulatory, antiviral, and antifungal properties (Juang and Liang, 2020; Yang Y. et al., 2021). Ginsenoside K regulates the expression of macrophage scavenger receptor class A1 (SR-A1), ATP Binding Cassette Subfamily A Member 1 (ABCA1), and ATP Binding Cassette Subfamily G Member 1 (ABCG1) through the NF-κB/MAPK signaling pathway, reduces lipid accumulation in macrophages, inhibits the transformation of macrophages to foam cells, and blocks ox-LDL-induced inflammatory responses and foam cell formation (Lu et al., 2020). *Gynostemma pentaphyllum* (Thunb.) Makino, regulates the expression of aortic cell apoptosis and oxidative stress-related proteins by activating the PI3K/AKT signaling pathway, while downregulated mitochondrial fission proteins results in anti-inflammatory effects and reduced inflammatory factor expression. This in turn reduces endothelial cell shedding and aortic intima thickening (Song N. et al., 2020). Andrographolide downregulates inflammatory factor levels by altering the phenotype of macrophages, thus inhibiting activation of the NF-κB pathway. Andrographolide improves endothelial cell systolic and diastolic dysfunction by reducing endothelin (ET)-1 and thromboxane (TX) A2, levels and increasing levels of NO and prostaglandin (PG) I2. It plays a role in delaying the inflammatory damage caused by coronary heart disease and in protecting arterial plaques (Shu et al., 2020). Paeoniflorin, is a terpene glycoside extracted from *Paeonia lactiflora* Pall. That has anti-inflammatory effects. It downregulates expression of the downstream regulatory protein myeloid differentiation primary response 88 (MyD88) in the TLR4 pathway, thus inhibiting the transcriptional activity of NF-kB and downregulating proinflammatory factor expression (Li et al., 2017). Paeoniflorin blocks the MAPK/NF-κB signaling pathway, then exerts anti-AS effects by inhibiting ox-LDL production during VSMC proliferation and migration, reducing inflammatory cytokine secretion and inhibiting foam cell formation (Li et al., 2018). Geniposide inhibits the polarization of M1 macrophages through the MAPK signaling pathway, which inhibits induction of inflammatory IL-1β and induces a phenotype similar to that of M2 macrophages under M1 differentiation conditions. Geniposide participates in M2 macrophage polarization by enhancing the expression of inflammatory factors such as Nuclear Receptor Subfamily four Group A Member 1 (NR4A1), cluster of differentiation 14 (CD14), and IL-10. It inhibits and stabilizes AS plaques (Jin et al., 2020). Geniposide combined with Panax notoginsenoside R1 inhibits the secretion of serum inflammatory and oxidative stress factors by inducing activation of the Nrf2 signaling pathway and synergistically reduces blood lipid levels and inhibits plaque formation (Liu et al., 2021). Other natural saponins, such as amygdalin (Wang et al., 2020), astragaloside IV(Zhang et al., 2022a), and saikosaponin (Yang et al., 2017) can also protect against AS plaques by modulating the MAPK/NF-κB signaling pathway and reducing inflammatory factors and oxidative stress responses.

### Quinones

Quinones comprise benzoquinone, naphthoquinone, phenanthrenequinone, and anthraquinone, all of which are ubiquitous in plants (Zhang L. et al., 2021). Emodin is an anthraquinone extracted from *Rheum palmatum* L. (Cui et al., 2020) that reduces ROS generation and attenuates homocysteine-activated MAPK phosphorylation. It also significantly increases the content of SOCS3 and decreases the contents of phosphorylated JAK kinase two and phosphorylated STAT, thus blocking the JAK/STAT signaling pathway and exerting anti-AS effects (Pang et al., 2015; Luo et al., 2021). Tanshinone IIA from *Salvia miltiorrhiza* Bunge is an active constituent of the diterpene phenanthrene quinone and has anti-inflammatory, antioxidant, and other biological effects (Xu et al., 2020). Tanshinone IIA might exert immunomodulatory, anti-inflammatory, and anti-AS effects by regulating the TLR4/MyD88/NF-κB signaling pathway (Chen et al., 2019).

## Conclusion and outlook

Atherosclerosis is the most prevalent type of arteriosclerotic vascular disease worldwide. It causes narrowing or even occlusion of the arterial lumen, which impedes the blood supply, resulting in ischemic pathological changes in corresponding organs (Wolf and Ley, 2019). The occurrence of AS is affected by many factors, among which inflammation and oxidative stress play crucial roles. The PI3K/AKT, TLR4, JAK/STAT, MAPK, and NF-κB pathways play roles in amplifying signaling during AS formation, and reducing the phosphorylation of the molecules in these pathways helps to alleviate the release of inflammatory factors, thus inhibiting the occurrence of AS. As the main antioxidant stress signaling pathway, Nrf2 plays a key role in restoring the physiological oxidative/antioxidative balance and protecting blood vessels from oxidative stress damage. Elevated intracellular Nrf2 levels can inhibit the pathological factors that mediate AS formation. Therefore, anti-inflammatory and antioxidant properties are crucial for AS treatment. Many natural medicines including TCM confer advantages and development prospects in terms of anti-inflammatory and anti-oxidative stress. This article described natural medicines including alkaloids, flavonoids, polyphenols, saponins, and quinones, all of which act on these pathways and confer beneficial therapeutic effects on AS. Most of them, including matrine, kaempferol, and resveratrol, act on several signaling pathways. We found that these natural drugs not only play an anti-AS role from anti-inflammatory and antioxidant perspectives but also inhibit the formation of vascular plaque and protect the intima of vessels by repairing the vascular endothelium, inhibiting the formation of foam cells, and controlling platelet activation. Table 1 shows the mechanisms of action through which natural medicines can help to treat AS.

**TABLE 1:** Regulatory effects of natural compounds of traditional Chinese medicine on AS-related signaling pathways.

Categories	Active ingredient	Plants	Extract method		Signaling pathways	Ref
			*In Vivo*	*In Vitro*		
Alkaloid	Cyclovirobuxinum D	*Buxus sinica var. Parvifolia* M. Cheng	–	RAW264.7 cells	JAK/STAT	Guo et al. (2014)
	Matrine	*Sophora flavescens* Aiton	–	HAVSMCs	NF-κB/MAPK	Wang et al. (2021)
			–	Macrophages	TLR4	Cui et al. (2022)
	Sinomenine	*Sinomenium acutum* (Thunb.)Rehder&E.H.Wilson	SD rats	–	TLR4/NF-κB	Geng et al. (2021)
	Ligustrazine	*Ligusticum chuanxiong* Hort	SD rats	–	NF-κB/Nrf2	Li Y. et al. (2019)
			SD rats	–	TLR4/NF-κB	Yang L. et al. (2021)
			SD rats	–	PI3K/AKT	Li L. et al. (2019)
	Berberine	*Coptis chinensis* Franch	ApoE^−/−^ mice	–	PI3K/AKT	Song and Chen, (2021)
Flavonoids	Kaempferol	*Centella asiatica* (L.) Urb	ApoE^−/−^ mice	–	PI3K/AKT/Nrf2	Feng et al. (2021)
	Quercetin	*Belamcanda chinensis* (L.) Redouté	–	HUVECs	NF-κB	Chen et al. (2020)
	Puerarin	*Puerariae Lobata* (Willd.) Ohwi	–	HUVECs	PI3K/AKT/NF-κB	Deng et al. (2017)
	Icariin	*Epimedium brevicornum* Maxim	Wistar rats	–	MAPK	Hu et al. (2016)
			SD rats	–	NF-κB/MAPK/Nrf2	Luo et al. (2022)
	Luteolin	*Clematis hexapetala* Pall	–	EA.hy926 cells	NF-κB	Jia et al. (2015)
			–	HUVECs	NF-κB/MAPK	Xia et al. (2014)
	Hydroxysafflor yellow A	*Carthamus tinctorius* L	ApoE^−/−^ mice	–	TLR4	Xue et al. (2021)
	Baicalin	*Scutellaria baicalensis* Georgi	ApoE^−/−^ mice	–	NF-κB/MAPK	Wu et al. (2018b)
	Wogonin		–	HUVECs	MAPK	Wu et al. (2019)
	Hesperetin	*Citrus reticulata* Blanco	Wistar rats	–	NF-κB/Nrf2	Olumegbon et al. (2022)
Polyphenols	Resveratrol	*Reynoutria japonica* Houtt	ApoE^−/−^ mice	–	PI3K/AKT	Ji et al. (2022)
			–	HAVSMCs	TLR4	Chen et al. (2022)
			–	HUVECs	JAK/STAT/TLR4/NF-κB	Zhang M. et al. (2019)
			–	HUVECs	NF-kB/MAPK	Pan et al. (2016)
	Curcumin	*Curcuma longa* L	ApoE^−/−^ mice	–	NF-κB	Lv et al. (2020)
	Theaflavine	*Camellia sinensis* (L.) Kuntze	ApoE^−/−^ mice	HUVECs	Nrf2	Zeng et al. (2021)
	Salvianolic acid B	*Salvia miltiorrhiza* Bunge	LDLR −/− Mice	–	NF-κB/MAPK	Zhang et al. (2022b)
	Catechin	*Camellia sinensis* (L.) Kuntze	–	Macrophages	NF-κB	Starzonek et al. (2019)
	Epicatechin		ApoE^−/−^ mice	–	NF-κB	Morrison et al. (2014)
	(-)-Epicatechin gallate		–	VSMCs	TLR4/NF-κB/MAPK	Li W. et al. (2021)
Saponins	Ginsenoside K	*Panax ginseng* C. A. Mey	–	RAW264.7 cells	NF-kB/MAPK	Lu et al. (2020)
	Gypenoside XVII	*Gynostemma pentaphyllum* (Thunb.) Makino	ApoE^−/−^ mice	–	PI3K/AKT	Song N. et al. (2020)
	Andrographolide	*Andrographis paniculata* (Burm. f.) Nees	C57BL/6 mice	–	NF-κB	Shu et al. (2020)
	Paeoniflorin	*Paeonia lactiflora* Pall	SD rats	–	TLR4/NF-κB	Li et al. (2017)
			–	VSMCs	NF-κB/MAPK	Li et al. (2018)
	Geniposide	*Gardenia jasminoides* J. Ellis	New Zealand rabbits	–	MAPK	Jin et al. (2020)
	Geniposide and Notoginsenoside R1	*Gardenia jasminoides* J. Ellis and *Panax notoginseng* (Burkill) F. H. Chen	ApoE^−/−^ mice	–	Nrf2	Liu et al. (2021)
	Amygdalin	*Eriobotrya japonica* (Thunb.) Lindl	ApoE^−/−^ mice	–	NF-κB/MAPK	Wang et al. (2020)
	Astragaloside IV	*Astragalus membranaceus var. mongholicus* (Bunge) P. K. Hsiao	LDLR −/− Mice	–	NF-κB/MAPK	Zhang et al. (2022a)
	Saikosaponin	*Bupleurum chinense* DC.	–	HAVSMCs	MAPK	Yang et al. (2017)
Quinones	Emodin	*Rheum palmatum* L	ApoE^−/−^ mice	–	JAK/STAT	Luo et al. (2021)
			–	VSMCs	MAPK	Pang et al. (2015)
	Tanshinone IIA	*Salvia miltiorrhiza* Bunge	ApoE^−/−^ mice	–	TLR4/NF-κB	Chen et al. (2019)

Natural medicines including TCM play multi-target, multi-path, and multi-linked regulatory roles in the treatment of AS. However, the nature of the investigative methodology and content remain problematic. The extraction and purification of natural medicines are relatively complex and affected by the environment, non-standardized products, medicinal material quality, and technological processes (Lefebvre et al., 2021). Traditional Chinese and other natural medicines do not rely on a single or specific type of active ingredient to exert therapeutic effects; they rather exert synergistic effects of numerous components acting on many pathways and targets. The pathogenesis of AS is complex and diverse, involving mechanisms of cytokine linkage to signaling pathways, cascade reactions of cytokines induced by these pathways, and interactions among signaling pathways (Libby, 2021). Therefore, the effects of natural medicines on these mechanisms and the intermediary mediators involved are important. Most mechanistic studies have focused on animal research and molecular biological approaches, whereas clinical research involving humans mostly remains at the level of blood sample analysis, and the methodology is relatively simple. However, AS is not easy to cure and the treatment cycle is long. Therefore, clinical studies of humans are needed to evaluate damage to metabolic organs, especially the liver and kidneys.

The formation of AS is the result of the cooperative actions of many mechanisms, and the inflammatory response and oxidative stress always run through the pathological formation of AS. TCM offers unique advantages for the prevention and treatment of AS. However, studies of most AS models are still in their infancy, and the upstream and downstream molecules in the pathways of some drug targets have not been specifically investigated. In addition, TCM exerts therapeutic effects through numerous targets and pathways, and formulae comprising several TCMs is the essence of TCM culture. This review explains only TMC compounds, which is one-sided. The mechanisms of TCM action and its compounds in the treatment of AS awaits discovery and validation by subsequent investigators. With the further development of biotechnology, such as bioinformatics analysis and network pharmacology (Fu et al., 2020; Zhou et al., 2020), an increasing number of active compounds with anti-AS effects in TCM will be discovered, providing more effective diagnostic tests and ideas for treating ASCVD using TCM.

## Glossary

### The following abbreviations are used in this document:

ABCA1: ATP-binding cassette transporter A1
ABCG1: ATP-binding cassette transporter G1
AKT: Proteinkinase B
AP-1: Activation protein-1
ARE: Antioxidant response element
AS: Atherosclerosis
ASCVD: Atherosclerotic Cardiovascular Disease
eNOS: Endothelial Nitric Oxide Synthases
ERK: Extracellular signal-regulated kinase
ET-1: Endothelin-1
GPER: G protein-coupled estrogen receptor
HASMCs: Human aortic smooth muscle cells
ICAM-1: Intercellular cell adhesion molecule-1
IHR: Intermittent hypoxia/reoxygenation
IL: Interleukin
iNOS: Inducible Nitric Oxide Synthase
IRFs: Interferon regulatory factors
JAK: Janus Kinase
JNK: c-Jun N-terminal protein kinase
LOX-1: lectin-like oxidized low-density lipoprotein re-ceptor-1
LPS: lipopolysaccharide
MAO: Monoamine oxidases
MAPK: Mitogen-Activated Protein Kinase
MDA: Malondialdehyde
MMP-9: Matrix metalloproteinase-9
MyD88: Myeloiddifferentiationfactor88
NF-κB: Nuclear Factor Kappa-B
NO: Nitric oxide
NR4A1: Nuclear Receptor Subfamily 4 Group A Member 1
Nrf2: Nuclear Factor E2-Related Factor2
ox-LDL: Oxidized low-density lipoprotein
p38MAPK: p38 mitogen-activated protein kinase
PGI2: Prostaglandin-I-2
PI3K: PhosphoInositide-3 Kinase
ROS: Reactive oxygen species
SOCS: Suppressors of cytokine signaling
SR-A1: Class A1 scavenger receptor
STAT: Signal Transducer And Activator Of Transcription
TCM: Traditional chinese medicine
TF: Tissue factor
TLR4: Toll Like Receptor 4
TNF-α: Tumor necrosis factor-α
TXA2: Thromboxane A2
VCAM-1: Vascular Cell Adhesion Molecule 1
VECs: Vascular endothelial cells
VEGF: Vascular endothelial growth factor
VSMCs: Vascular smooth muscle cells
WHO: World Health Organization.
